# Sub-Inhibitory Concentrations of Amoxicillin and Tylosin Affect the Biofilm Formation and Virulence of *Streptococcus suis*

**DOI:** 10.3390/ijerph19148359

**Published:** 2022-07-08

**Authors:** Jing Zuo, Qingying Fan, Jinpeng Li, Baobao Liu, Bingqian Xue, Xiaoling Zhang, Li Yi, Yang Wang

**Affiliations:** 1 College of Animal Science and Technology, Henan University of Science and Technology, Luoyang 471000, China; z4379479232021@163.com (J.Z.); fanqingyingbaby@163.com (Q.F.); 151413140107@stu.haust.edu.cn (J.L.); babyliu8242@163.com (B.L.); xbq_6666@163.com (B.X.); zjl926@163.com (X.Z.); 2 Key Laboratory of Molecular Pathogen and Immunology of Animal of Luoyang, Luoyang 471000, China; 3 College of Life Science, Luoyang Normal University, Luoyang 471000, China

**Keywords:** antibiotics, biofilm, *Streptococcus suis*, sub-inhibitory concentration, virulence

## Abstract

*Streptococcus suis* (*S. suis*) can form a protective biofilm during infection and lead to prolonged disease. Oral antibiotics are often used for treatment in clinical practice, but sub-inhibitory concentration levels often exist due to low oral absorption rate, resulting in disease deterioration. The purpose of this study was to investigate the effects of Amoxicillin and Tylosin on the biofilm formation and virulence of *S. suis* HA9801 at sub-inhibitory concentration. We first determined that the test groups (1/4MIC Amoxicillin and Tylosin) could significantly increase the amount of biofilm formation without affecting bacterial growth. The LD50 value of the test groups was significantly higher than that of the control group in the mouse infection model. In the mouse infection model, the LD50 value of the experimental group was significantly increased, but the tissue bacterial load was significantly decreased. Further RT-PCR analysis showed that the expression levels of virulence-related genes in the experimental group were significantly reduced. Our study suggests that both Amoxicillin and Tylosin at sub-inhibitory concentrations could enhance the biofilm formation ability of *S. suis* HA9801 and reduce its virulence to form persistent infection.

## 1. Introduction

*Streptococcus suis* (*S. suis*) is an important zoonotic agent with an extensive distribution worldwide [1]. It can cause substantial economic losses in the pig industry [2,3] and cause systemic infections in humans [4], which seriously endangers human health. The data show that *S. suis* is also a significant public health problem in east Asia and southeast Asia. Since the discovery, in 1968, that an infection of *S. suis* serotype 2 could cause human meningitis, more than 1600 human cases in Asian have been recorded [5]. It has been proven that *S. suis* can cause persistent infections that are difficult to eradicate with antibiotics because it forms a biofilm in the body [6]. Studies have shown that the formation of bacterial biofilms may be affected by virulence factors, temperature, pH, antibiotics, and other factors [7]. Among them, the use of sub-concentration antibiotics is a critical reason for promoting the formation of bacterial biofilms. It has been reported that the average annual consumption of veterinary antibiotics has reached approximately 6000 tons in China [8]. A survey on antibiotics in pig farms in Thailand shows that Amoxicillin is the most commonly used antibiotic. The total amount of antibiotics mixed into pig medicated feed in 2017 is estimated to be 843 tons [9]. Although the total amount of antibiotics used on farms is significant, due to the low oral absorption rate, irregular medication, and illegal use of low-dose antibiotic growth promoters, antibiotics often exist in livestock and poultry at sub-inhibitory concentration levels [10], which leads to prolonged disease and treatment difficulties. Oral antibiotic powders (Amoxicillin, lincomycin, oxytetracycline, doxycycline, gentamicin, and Tylosin, etc.) are often the first choice for pig farms to treat *S. suis* from the perspective of clinical treatment prescriptions on farms [11,12].

A study found that exposure to sub-inhibitory concentrations of lincomycin and oxytetracycline increased the biofilm formation of *S. suis* [13]. *Pseudomonas aeruginosa* (*P. aeruginosa*) also showed similar results after the use of sub-inhibitory concentrations of cefotaxime, Amoxicillin, and azithromycin [14]. Worryingly, *Escherichia coli* (*E. coli*) will form biofilms at lower the inhibitory concentration of olaquindox (OLA), which can lead to drug resistance [15]. Additionally, studies have shown that a sub-inhibitory concentration of antibiotics may affect the virulence genes of bacteria, such as Panton-Valentine-leucocidin (PVL), alpha-hemolysin (Hla), and phenol-soluble-modulin alpha (PSMα) [16,17]. The previous experiments of our research group confirmed this view [18]. Norfloxacin can significantly down-regulate the key virulence genes of *S. suis*, such as capsular polysaccharide (CPS), extracellular factor (EF), hemolysin (SLY), fibronectin and fibrinogen binding protein (FBPS), glutamate dehydrogenase (GDH), and glycerophosphate deoxygenase (GAPDH). Similarly, when *E. coli* was treated with a sub-inhibitory concentration of piperacillin-tazobactam, a significant reduction in virulence was detected in a mouse model of abdominal infection by tissue carrier assay [19]. We have similar findings in *S. suis*. Norfloxacin at sub-inhibitory concentrations could enhance the ability of *S. suis* biofilm formation, increase the number of viable bacteria in the biofilm, and significantly reduce the expression levels of virulence factors *ef*, *fbps*, *gapdh*, *gdh*, *mrp*, and *gapdh*, resulting in persistent infection [18]. As commonly used drugs for the treatment of swine streptococcus, it is currently unknown whether Amoxicillin and Tylosin can affect the formation of *S. suis* biofilm and regulate virulence at sub-inhibitory concentrations.

The aim of this study was to explore the effects of Amoxicillin and Tylosin on the biofilm formation and virulence of *S. suis* HA9801 at sub-inhibitory concentrations. We compared the growth kinetics, biofilm formation, virulence, and histopathological damage of the control group and the drug group by adding sub-inhibitory concentrations of Amoxicillin and Tylosin.

## 2. Materials and Methods

### 2.1. Ethics Approval Information

The animal study protocol was approved by the Animal Care and Use Committee of Henan University of Science and Technology (approval number: SKKUIACUC-18-04-14-3, date of approval: 14 April 2018).

### 2.2. Bacterial Strains and Antibiotics

The *S. suis* HA9801 strain was isolated from a sick pig during an epidemic outbreak in 1998 in Jiangsu, China, and it was confirmed to be a virulent strain. Under normal conditions, the *S. suis* HA9801 strains were grown at 37 °C in tryptic soy broth (TSB; HuanKai biology, Guangzhou, China) or on tryptic soy agar (TSA; HuanKai biology, Guangzhou, China) plates with 10% (*v*/*v*) newborn bovine serum (NBS; TSB; YeYuan biology, Shanghai, China). Amoxicillin and Tylosin bulk drugs were purchased from the China Institute of Veterinary Drugs Control (Beijing, China), and antibiotic solutions were prepared according to the content. Stock solutions with a concentration of 1280 μg/mL of all antibiotics were used.

### 2.3. Determination of MIC and MBC

The MICs toward *S. suis* HA9801 of Amoxicillin and Tylosin were detected by the broth microdilution method according to the American Committee for Clinical and Laboratory Standards Institute methods (CLSI) [20]. Briefly, a volume of 100 µL of TSB broth was transferred to each well of a 96-well microtiter plate. Amoxicillin and Tylosin in a concentration range from 0.15625 to 80 μg/mL were added to 96-well plates containing TSB medium by the multiple dilution method. *S. suis* HA9801 was cultured to the logarithmic growth phase, diluted to a concentration of 1 × 10^5^ CFU/mL, then transferred to each microwell in a 1:1 ratio and cultured statically at 37 °C for 24 h. TSB medium was used as the negative control group, with three wells for each treatment as parallel samples. The required data were read through naked-eye observation, and the lowest drug concentration that could completely inhibit the growth of bacteria in the wells was the MIC. After the antibacterial test, the wells were thoroughly cleaned with 0.9% NaCl to remove any antibacterial residues. Fresh 200 μL TSB growth medium was added to the cleaned wells and incubated for 24 h at 37 °C. The plates were inspected for bacterial growth. The MBC refers to the minimum drug concentration required to kill 99.9% of the tested microorganisms.

### 2.4. Biofilm Formation Assay

The biofilm formation of *S. suis* HA9801 was determined using the crystal violet semi-quantitative staining described by Christensen et al., with some modifications. The strain, cultured in TSB medium for 8 h to 1 × 10^6^ CFU/mL, was diluted. The 96-well plate (Costar, Corning, NY, USA) was inoculated with or without 1/4MIC Amoxicillin (0.078125 μg/mL) or 1/4MIC (0.3125 μg/mL) Tylosin. TSB medium without bacteria was used as the negative control. After the bacteria were incubated at 37 °C for 24 h, the medium and free-floating bacteria were removed. The planktonic bacteria were washed off using sterile phosphate-buffered saline (PBS). They were then fixed in methanol for 20 min, stained with 0.1% ammonium oxalate crystal violet for 15 min, washed lightly and dried, and finally dissolved in 95% ethanol for 30 min, and the optical density was measured at 595 nm with a full wavelength microplate reader (Synergy2, Biotek, Vermont, NY, USA). The experiments were run in triplicate.

### 2.5. Growth Curve

The effects of sub-concentration antibiotics on the growth rate of *S. suis* HA9801 were measured. *S. suis* HA9801 was inoculated in the TSB medium. After overnight culture, the suspended bacteria fluid was supplemented with Amoxicillin and Tylosin at a final concentration of 1/2MIC, 1/4MIC, 1/8MIC, and 1/16MIC, respectively, and incubated at 37 °C with constant shaking. The optical density at 600 nm (OD_600_) was measured by UV spectrophotometer at 1, 2, 3, 4, 5, 6, 7, 8, 9, 10, 12, and 14 h. The growth curve was constructed by plotting the absorbance versus the incubation time [21].

### 2.6. Confocal Laser Scanning Microscopy (CLSM)

Biofilm formation of *S. suis* HA9801 under sub-concentration of Amoxicillin (0.078125 μg/mL) and Tylosin (0.3125 μg/mL) was evaluated using live/dead bacterial viability kits (LIVE/DEAD BacLight Bacterial Viability Kit L13152, Thermo Fisher Scientific, Beijing, China). Biofilms of *S. suis* HA9801 were cultured according to method 2.3, and cell slides were placed in 6-well plates and incubated at 37 °C for 24 h. The biofilm attached to the cell slides was gently washed with sterile PBS, then stained with a backlight live/dead viability kit consisting of Syto9 and propidium iodide (PI). Following staining, they were washed with distilled water and dried at room temperature. The samples were observed by laser confocal scanning microscope (Zeiss LSM CLSM); 488 nm laser excitation shows a green fluorescence emission of live bacteria, while 543 nm laser excitation excites a red fluorescence emission of bacteria whose membrane was damaged.

### 2.7. LD50 Assay

To test the virulence of *S. suis* HA9801 strains and 1/4MIC Amoxicillin and Tylosin-treated strains, the median lethal dose was determined. The strain grew to the exponential phase (8 h) and was collected and washed twice with normal saline, and then diluted from 10^11^ CFU to 10^4^ CFU, according to the results of previous studies in the experimental group; each group of mice were given the appropriate challenge dose. Ten 4-week-old SPF female BALB/C mice were intraperitoneally injected in each group. The mortality rate was monitored until 14 days after infection. The results were averaged and calculated using the method of Reed and Muench.

### 2.8. Detection of Bacterial Viscera Distribution in Mice

*Streptococcus suis* was grown overnight at 37 °C and then diluted with normal saline to a concentration of 5 × 10^6^ CFU/mL. Fifteen 4-week-old SPF female BALB/C mice were injected intraperitoneally with bacterial fluid to induce infection [22]. Mice infected with *Streptococcus suis* were randomly divided into 3 groups of 5 mice each. Treatments by intraperitoneal injection included: Group 1: Vehicle control group; Group 2: 1/4 MIC Amoxicillin; Group 3: 1/4 MIC Tylosin. The mice were euthanized at 12 h after infection, and the lung, liver, and spleen were removed and homogenized in PBS containing 20% glycerol. After appropriate dilution, the bacteria were applied to the TSA plate for counting and CFU/mL expression. The count range was 30–300.

### 2.9. Histological Examination

The *S. suis* HA9801 was treated with 1/4MIC Amoxicillin and Tylosin; after 12 h intraperitoneal injection, the lungs, liver, and spleen were taken out from the euthanized mice. They were fixed in 4% formaldehyde at 4 °C for 12 h, and then embedded in paraffin. The paraffin blocks were then cut into 4 μm sections and stained with hematoxylin and eosin and inspected with an optical microscope (Axiophot; Zeiss, Oberkochen, Germany). CCIZ soft imaging system and analysis research software was used to capture and process digital images.

### 2.10. RNA Extraction and Quantitative Reverse Transcription Polymerase Chain Reaction (qRT-PCR)

In order to investigate the effect of sub-MICs of Amoxicillin and Tylosin on the expression of virulence-related genes, after *S. suis* HA9801 grew to the mid-log phase, Amoxicillin and Tylosin were added at 1/4MIC before further incubation at 37 °C for 12 h, and normal saline was added for the control group. The bacteria were collected, and RNA was extracted using a TRIzon-RNA Extract kit (Invitrogen TRIzol, Thermo Fisher Scientific, Carlsbad, CA, USA), according to the manufacturer’s instructions. Reverse transcription was carried out using a first-strand cDNA synthesis kit (Cwbio, Taizhou, China), with random primers. The reverse transcription conditions were 42 °C for 15 min and 85 °C for 5 min. For real-time PCR (RT-PCR) mRNA expression measurements, quantitative specific primers *gdh*, *mrp*, *gapdh*, *sly*, *fbps*, *ef*, and *cps2a* were purchased from Azenta (Azenta Life Sciences, Suzhou, China). PCR conditions included an initial denaturation at 95 °C for 30 s, followed by 40 cycles of denaturation at 95 °C for 10 s, and annealing at 60 °C for 30 s. Melting curve analysis was then performed. The 16S rRNA gene was chosen as the internal control. All experiments were performed in triplicate. In order to evaluate the specificity of amplification, a melting curve analysis was plotted at the end of each PCR run. The relative expression level was measured with 2^−ΔΔCt^ method [23]. ΔΔCt = ΔCt (test sample) − ΔCt (reference sample), ΔCt = Ct (target gene) − Ct (internal reference gene).

### 2.11. Statistical Analysis

Graph 8.0 was used to perform statistical analysis on the data. One-way analysis of variance (ANOVA) was used for the data, and *p* < 0.05 was considered statistically significant.

## 3. Results

### 3.1. MIC and MBC of Amoxicillin and Tylosin for S. suis HA9801

The MICs of Amoxicillin and Tylosin on the field isolates of selected *S. suis* HA9801 strains, as listed in Table 1, were 0.3125 and 1.25 μg/mL, and the MBCs were 2.5 and 1.25 μg/mL, respectively.

### 3.2. Effect of Sub-MIC Amoxicillin and Tylosin on Biofilm Formation

**Figure 1:**
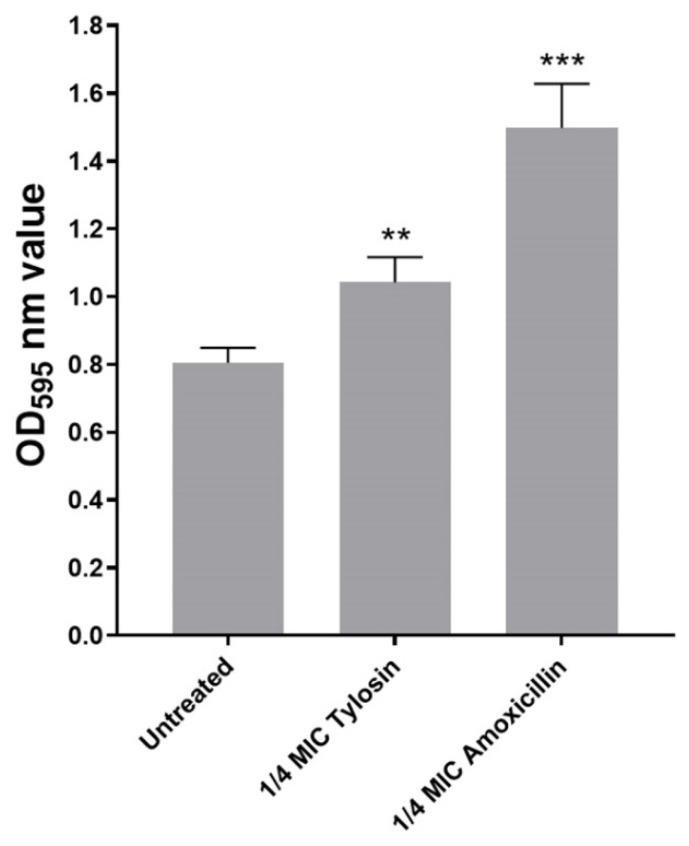
Biofilm formation of *S. suis* HA9801 growing at 1/4MIC Amoxicillin and 1/4MIC Tylosin. Biofilm formation was determined following staining with crystal violet. The optical density (OD) of 1/4MIC Amoxicillin and 1/4MIC Tylosin were measured at 595 nm. The statistical analysis used was one-way analysis of variance. ** *p* < 0.01, and *** *p* < 0.001.

In this study, we explored sub-MIC antibiotics’ effects on biofilm formation using a standard crystal violet assay. As shown in Figure 1, we found that, compared with the control group without antibiotics, 1/4MIC Amoxicillin and Tylosin showed a significant increase (*p* ≤ 0.01) in biofilm formation ability.

### 3.3. Growth Curve

**Figure 2:**
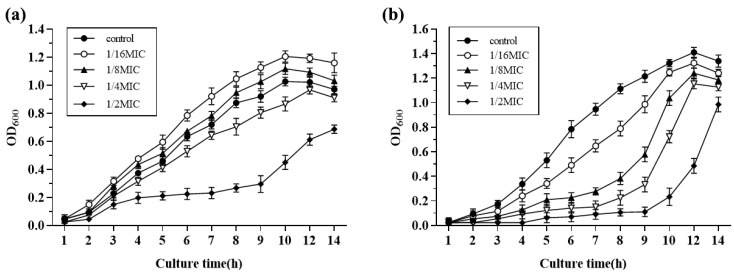
The growth curves of *S. suis* HA9801 in the presence of sub-concentrations of Amoxicillin and Tylosin are presented. (**a**) Growth curves of *S. suis* HA9801 in the presence of sub-concentration Amoxicillin. (**b**) Growth curves of *S. suis* HA9801 in the presence of sub-concentration Tylosin. 1/2MIC Amoxicillin and Tylosin significantly inhibited the growth of *S. suis*. The growth of *S. suis* treated with 1/4MIC, 1/8MIC, and 1/16MIC was inhibited at 4–10 h, but there was no significant difference after entering the plateau stage (12 h).

Growth curves of *S. suis* HA9801 at sub-inhibitory concentrations of Amoxicillin and Tylosin are presented in Figure 2; 1/2MIC Amoxicillin and Tylosin significantly inhibited the growth of *S*. *suis*. The growth of *S. suis* HA9801 treated with 1/4MIC, 1/8MIC, and 1/16MIC was inhibited at 4–10 h, but there was no significant difference after entering the stationary phase (12 h).

### 3.4. Confocal Laser Scanning Microscopy (CLSM)

**Figure 3:**
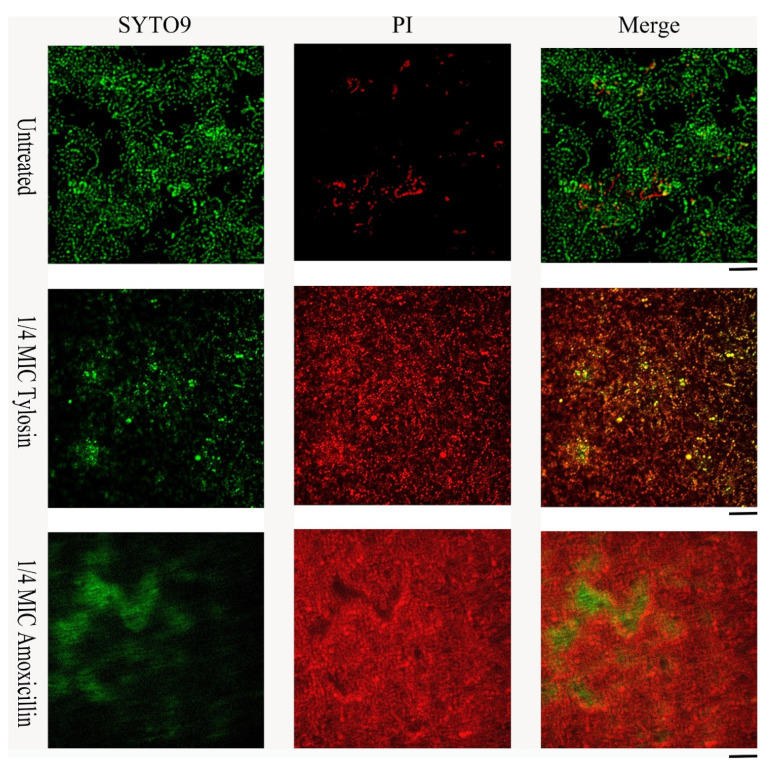
Effects of sub-concentration Amoxicillin (0.078125 μg/mL) and Tylosin (0.3125 μg/mL) on the formation of *S. suis* HA9801 biofilm at 24 h. *S. suis* HA9801 biofilms were stained using live and dead bacterial viability kits and analyzed using a confocal microscope. Bacteria with intact cell membranes were stained green, while bacteria with damaged cell membranes were stained red. Live (**left**), dead (**middle**), and merged (**right**) are present in biofilms without antibiotics, 1/4MIC Amoxicillin, and 1/4MIC Tylosin stimulation.

Live/dead cell staining was performed for *S. suis* HA9801 biofilm formation in conditions with 1/4MIC Amoxicillin and Tylosin (Figure 3). The control biofilm showed abundant live colonies, but no aggregation was observed. The results stimulated by 1/4MIC Amoxicillin and Tylosin showed significant colony aggregation, and merged images indicated that the dead colonies encapsulated the living colonies. These results suggest that after using sub-concentrations of Amoxicillin and Tylosin, the degree of biofilm aggregation was significantly higher than that of the control group.

### 3.5. LD_50_ Assay

The virulence of *S. suis* HA9801, 1/4MIC Amoxicillin, and Tylosin-treated strains were determined in mouse models. Mice were infected with various doses of the strain, and their mortality was observed after 14 days. As shown in Table 2, the LD_50_ value was 2.245 × 10^6^ CFU for *S. suis* HA9801, and the LD_50_ values of the cells treated with 1/4MIC Amoxicillin and Tylosin were 100 times higher than *S. suis* HA9801, which were 2.198 × 10^8^ CFU and 1.763 × 10^8^ CFU, respectively.

### 3.6. Bacterial Distribution of Amoxicillin and Tylosin at Subminimum Inhibitory Concentrations

**Figure 4:**
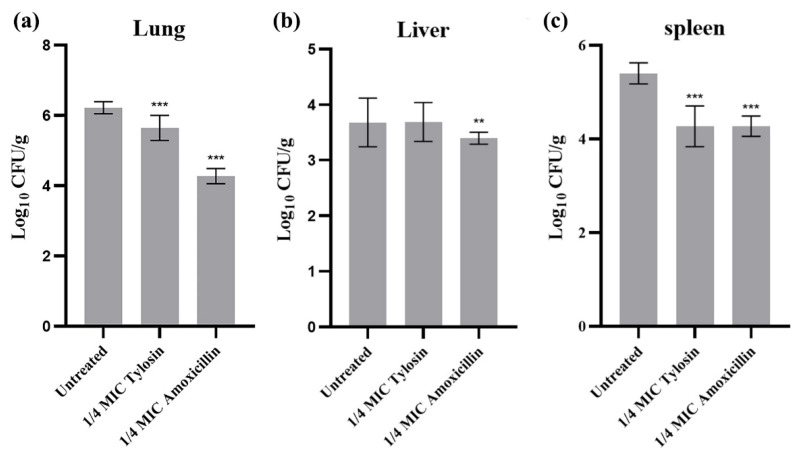
Bacterial counts in different organs after infection. (**a**) Bacterial counts in the lung after infection with wild strains, 1/4MIC Amoxicillin, and 1/4MIC Tylosin. (**b**) Bacterial counts in the liver after infection with wild strains, 1/4MIC Amoxicillin, and 1/4MIC Tylosin. (**c**) Bacterial counts in the spleen after infection with wild strains, 1/4MIC Amoxicillin, and 1/4MIC Tylosin. Mice were euthanized 12 h after injection. The sample was continuously diluted with a TSA plate, and the mean standard error for each sample of viable strains was calculated as colony formation units/g. The amount of bacteria in the tissues and viscera of mice treated with 1/4MIC Amoxicillin and Tylosin was significantly different from that of the HA9801 group. ** *p* < 0.01, and *** *p* < 0.001.

To further evaluate the effects of sub-concentrations of Amoxicillin and Tylosin on the pathogenicity of *S. suis* HA9801, we determined the differences of viable bacteria in organs using intraperitoneal inoculation. The bacterial loads of the lung (Figure 4a), liver (Figure 4b), and spleen (Figure 4c) were measured at 12 h after infection. The results showed that the bacterial loads in the lung, liver, and spleen of mice treated with 1/4MIC Amoxicillin and Tylosin were significantly lower than those in the HA9801 group (*p* < 0.01).

### 3.7. Histological Analysis

**Figure 5:**
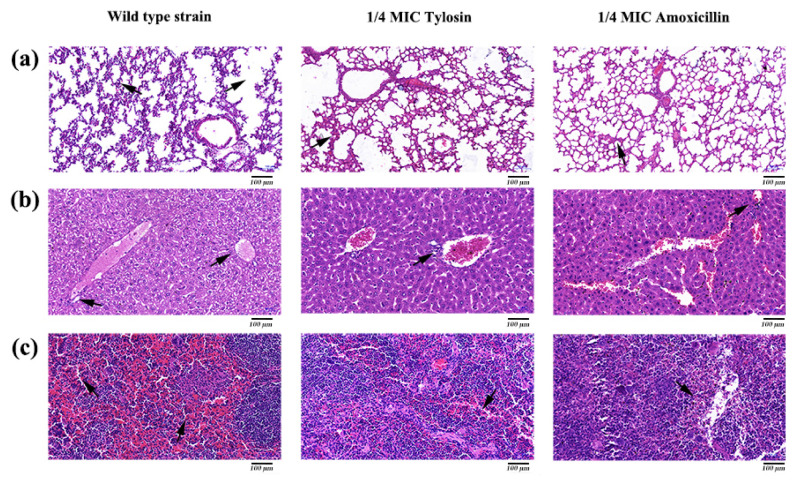
Histopathological observation of mice caused by *S. suis* HA9801 treated with 1/4MIC Amoxicillin and Tylosin. (**a**) Histopathological observation of the lung mice in after infection with wild strains, 1/4MIC Amoxicillin, and 1/4MIC Tylosin. (**b**) Histopathological observation in the liver of mice after infection with wild strains, 1/4MIC Amoxicillin, and 1/4MIC Tylosin. (**c**) Histopathological observation in the spleen of mice after infection with wild strains, 1/4MIC Amoxicillin, and 1/4MIC Tylosin. Scale bars: 100 µm.

Histopathological analysis was performed by HE staining. The results showed that the lungs, liver, and spleen in the *S. suis* HA9801 group showed obvious histological changes (Figure 5), such as alveolar congestion, punctate hepatocyte necrosis, and spleen congestion. Still, only slight inflammatory lesions were found in the tissues and organs in the 1/4MIC Amoxicillin and Tylosin groups. Tissue HE staining showed that normal lung tissue structure disappeared in the *S. suis* HA9801 group, and there was alveolar hyperemia and pulmonary septum widening (Figure 5a). There were large vacuoles in the cytoplasm of liver cells, necrosis of liver cells, local infiltration of inflammatory cells, destruction of hepatic cord structure, and infiltration of inflammatory cells in the portal duct area (Figure 5b). The spleen showed red bone marrow sinus dilatation, ferrofuscin deposition, and abundant red blood cell (Figure 5c).

### 3.8. Expression Profiling of Virulence Genes

**Figure 6:**
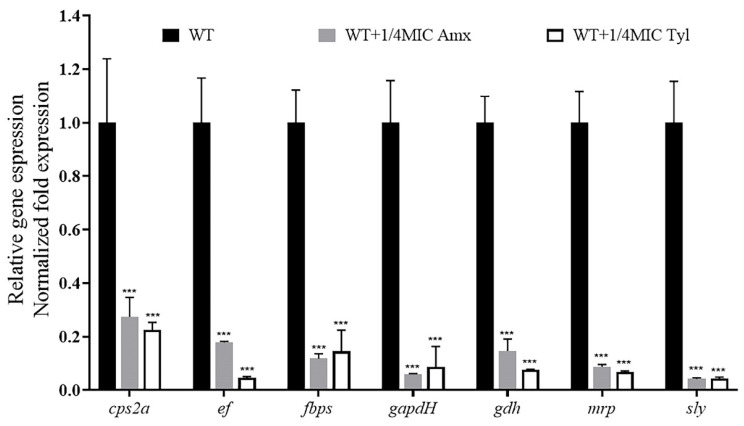
Effects of sub-concentration antibiotics on virulence-related gene expression of *S. suis* HA9801. Cultivation of *S. suis* HA9801 with exponential growth, without antibiotics or treatment with 1/4MIC Amoxicillin and Tylosin for 12 h. A sample of each culture was taken, the OD_600_ was adjusted to 1, then random primers were used for total RNA extraction and subsequent reverse transcription, as described above. The obtained cDNA serves as a template, and specific *ef*, *fbps*, *gapdh*, *gdh*, *mrp*, and *sly* primers were used for light cycler PCR amplification. The results showed that the mRNA expression levels of virulence factors *ef*, *fbps*, *gapdh*, *gdh*, *mrp*, and *sly* were significantly decreased in the 1/4MIC Amoxicillin and Tylosin-treated strains compared with that without antibiotic treatment. (*p* < 0.001). *** *p* < 0.001.

We explored the effects of sub-concentrations of Amoxicillin (0.078125 μg/mL) and Tylosin (0.3125 μg/mL) on the mRNA expression levels of virulence-related genes in *S. suis* HA9801. The expression levels of the genes *cps2a, ef*, *fbps*, *gapdh*, *gdh*, *mrp*, and *sly* were quantified by qRT-PCR. In the exponential phase, total RNA was extracted, and the OD_600_ value was between 0.6 and 0.8. The results are shown in Figure 6; the mRNA expression levels of virulence factors *cps2a, ef*, *fbps*, *gapdh*, *gdh*, *mrp*, and *sly* were significantly decreased in the treatment of 1/4MIC Amoxicillin and Tylosin compared with the strain of *S. suis* HA9801 without antibiotics.

## 4. Discussion

*Streptococcus suis* (*S. suis*) is an important zoonotic pathogen that often causes persistent infection in clinical practice, seriously endangers human health, and causes huge economic losses to the pig industry [2,3]. Bacterial persistent infection is caused by many factors, such as bacterial resistance, biofilm formation, and the emergence of intracellular niches [24,25]. The formation of biofilm is an important factor among them. Preliminary studies in this laboratory have shown that *S. suis* is often colonized in pigs in the form of biofilms, resulting in repeated infections and difficulty to control [26]. Studies have found that sub-MIC antibiotics can enhance the ability of bacteria to form biofilms and cause persistent infections [27]. Clinically, the most commonly used method for early prevention or treatment of animal-related bacterial diseases is to add oral antibiotics to water or feed. However, due to pharmacokinetics/pharmacodyamics, antibiotics are mostly present in animals as sub-MICs [28]. In Southeast Asia, such as in China, Vietnam, Cambodia, and other developing countries, Amoxicillin and Tylosin are often used as feed additives at sub-inhibitory concentrations to prevent *S. suis* infection [29]. Therefore, exploring the effects of sub-inhibitory concentrations of Amoxicillin and Tylosin on the biofilm formation and virulence of *S. suis* can provide a reference for the correct clinical use of these antibiotics to prevent persistent bacterial infections, and guide the rational use of antibiotics by clinical veterinarians to avoid the increase in bacterial resistance and environmental antibiotic pollution caused by mistakes or the abuse of antibiotics.

Through in vitro MIC and MBC tests, we found that *S. suis* HA9801 is sensitive to Amoxicillin and Tylosin. However, this result is contrary to the case of clinical swine streptococcus disease in pig farms, which arouses great concern. Combined with the actual situation of the abuse of sub-concentration antibiotics as growth promoters, we further tested the effect of sub-concentration Amoxicillin and Tylosin on the biofilm formation ability of *S. suis*. The results of crystal violet semi-quantitative staining showed that 1/4MIC Amoxicillin and Tylosin could significantly increase the amount of biofilm formation without affecting the growth of *S. suis* HA9801 (Figure 1). This is consistent with the views of Wang et al., who reported that the use of sub-inhibitory concentrations of Tylosin would lead to a significant increase in the formation of biofilm, thereby enhancing the resistance of *S. suis* [30]. We further observed the biofilm with CLSM, and the results showed that after using the sub-concentration of Amoxicillin and Tylosin, the degree of biofilm aggregation was significantly higher than that of the control group. Hathroubi et al. also observed that the sub-MIC penicillin G in *Actinobacillus pleuropneumoniae* (*A. pleuropneumoniae*) significantly affected biofilms, increased biomass and biofilm thickness, and induced phenotypic changes in biofilm structure [31]. Similarly, the study by Rafaque et al. showed that, in the presence of sub-inhibitory concentrations of ciprofloxacin, the biofilm formation ability of *Escherichia coli* (*E. coli*) was significantly increased [32]. Moreover, Mlynek et al. found that sub-inhibitory concentrations of Amoxicillin can induce the production of extracellular genes in *Staphylococcus aureus* (*S. aureus*), thereby stimulating the formation of biofilms [33]. Under adverse external environments, such as ultraviolet rays and various disinfectants and antibiotics, bacterial aggregation is a self-defense mechanism used by bacteria that can increase survival and persistence in the body [34].

In fact, biofilm formation is now recognized as an important "virulence factor" for bacteria, making bacteria develop significant resistance to a variety of chemical, physical, and biological antimicrobial agents and is one of the main causes of persistent infection. Therefore, we determined that sub-concentrations of Amoxicillin and Tylosin affected the LD_50_ of mice infected with *S. suis* HA9801 and monitored the degree of damage to multiple organs in the infected mice. The LD_50_ results (Table 2) show that the lethality of the 1/4MIC Amoxicillin and Tylosin group of mice was significantly lower than that of the control group.

The tissue organ distribution (Figure 4) showed that, after treatment with 1/4MIC Amoxicillin and Tylosin, the amount of bacteria in the lung, liver, and spleen of the mice was significantly lower than that in the control group. We speculate that this may be caused by the down-regulation of virulence factors by sub-concentration antibiotics [35]. Furthermore, the results of tissue HE staining (Figure 5) showed that, after treatment with 1/4MIC Amoxicillin and Tylosin, respectively, the damage degree of each tissue was significantly lower than that of the control group, which indicates that 1/4MIC Amoxicillin and Tylosin might be involved in the expression of multiple genes that regulate bacterial virulence [36]. We selected seven virulence-related genes in *S. suis* for verification, which were *ef*, *fbps*, *gapdh*, *gdh*, *mrp*, and *sly* [18]. Compared with the control group, 1/4MIC Amoxicillin and Tylosin can significantly down-regulate the gene expression of *ef*, *fbps*, *gapdh*, *gdh*, *mrp*, and *sly*. The above seven genes exist in a wide range of *S. suis* and play a role in the virulence of *S. suis*, such as cell attachment and immune evasion. Studies have shown that sub-inhibitory concentrations of ceftazidime can promote the production of *Clostridium difficile* (*C. difficile*) toxins by affecting the expression level of *C. difficile* virulence genes, resulting in persistent infections [37]. Research by Grundstad et al. showed that vancomycin at sub-inhibitory concentrations could modulate the virulence of *Methicillin-resistant Staphylococcus aureus* (MRSA) [38]. Our results suggest that 1/4MIC Amoxicillin and Tylosin can significantly reduce the transcription level of these genes and reduce the body’s immune response, which indirectly promotes the formation of biofilms and the enhancement of bacterial resistance, posing a threat to the ecological environment. However, it is unclear whether this conclusion is general, that is, whether all sub-concentrations of antibiotics can cause any bacteria to develop biofilms and increase virulence. In view of this, future research could explore the biofilm formation ability and virulence changes of important pathogens under various sub-concentration antibiotic stress.

## 5. Conclusions

Our findings suggest that 1/4MIC Amoxicillin and Tylosin significantly up-regulate the biofilm-forming ability of *S. suis* HA9801 and down-regulate its virulence in mic, and indicate that 1/4MIC Amoxicillin and Tylosin can indirectly cause persistent infection of *S. suis* HA9801 in mice. This experiment may provide a theoretical basis for the development of future antibiotic treatment methods. It also provides some indications for the rational use of antibiotics in our clinic, thereby reducing the threat to the ecological environment and the global impact of AMR.

## Figures and Tables

**Table 1:** MICs and MBCs of Amoxicillin and Tylosin against *S. suis*.

Antibiotics	MIC (μg mL^−1^)	MBC (μg mL^−1^)
Amoxicillin	0.3125	2.5
Tylosin	1.25	1.25

**Table 2:** LD_50_ of Amoxicillin and Tylosin at sub-minimum inhibitory concentration.

Group	CFU Per Mouse				Mortality				LD_50_/(CFU)
WT	2.587 × 10^7^	3.014 × 10^6^	2.601 × 10^5^	3.532 × 10^4^	9/10	8/10	5/10	3/10	2.245 × 10^6^
1/4MIC Amoxicillin	2.313 × 10^10^	2.141 × 10^9^	2.162 × 10^8^	3.142 × 10^7^	8/10	6/10	5/10	4/10	2.198 × 10^8^
1/4MIC Tylosin	2.121 × 10^10^	3.012 × 10^9^	2.145 × 10^8^	3.114 × 10^7^	9/10	8/10	4/10	4/10	1.763 × 10^8^

## Data Availability

The original contributions presented in the study are included in the article; further inquiries can be directed to the corresponding authors.
